# Enhanced effect of checkpoint inhibitors when given after or together with IMM-101: significant responses in four advanced melanoma patients with no additional major toxicity

**DOI:** 10.1186/s12967-018-1602-8

**Published:** 2018-08-14

**Authors:** Angus G. Dalgleish, Satvinder Mudan, Alberto Fusi

**Affiliations:** 1 Infection & Immunity Research Center, St George’s University of London, Cranmer Terrace, London, SW17 0RE UK; 2 St George’s University of London, Imperial College, London, UK; 3 The Royal Marsden Hospital, London, UK; 4 Charité Comprehensive Cancer Center, Berlin, Germany

**Keywords:** Checkpoint blockade, Melanoma, IMM-101, Immunotherapy, *Mycobacteria*

## Abstract

**Background:**

The use of checkpoint inhibitors (ipilimumab, pembrolizumab, nivolumab) has revolutionised the treatment of metastatic melanoma. However still more than the half the patients do not respond to single-agent immunotherapy. This has led to the development of combining these agents in an attempt to enhance the anti-cancer activity. More than 300 different studies with 15 different drug doses are currently ongoing. Combining different checkpoint inhibitors (CPIs) does indeed lead to an increase in response rate, but this is associated with significant toxicity. IMM-101 is a heat killed Mycobacterium preparation which induces marked immune modulation and little systemic toxicity. It has been reported as having activity in melanoma as single agent and in pancreatic cancer in combination with gemcitabine, the latter in a randomised study.

**Methods:**

Here we report the effect of adding CPIs to 3 patients who had previously been on IMM-101, either as a trial or a named patient programme and a patient who received the IMM-101 together with nivolumab.

**Results:**

All 4 patients had rapid and very good responses, three of them maintained over 18 months with no significant additional toxicity.

**Conclusions:**

The rapid and complete clinical responses seen in these patients may suggest that IMM-101 is activating a complementary pathway which is synergistic with CPI treatment.

## Background

Immune checkpoint blockades or checkpoint inhibitors (CPIs) have emerged as a major breakthrough for patients with metastatic melanoma and more recently they have been licensed in other tumour types. Ipilimumab is an anti-CTLA-4 antibody which showed to significantly improve long term survival of patients with metastatic melanoma being up to 20% of the treated patients alive at 5 years in spite of a low response rate (overall response rate [ORR] is around 10% with a complete response [CR] rate of less than 3%) [1]. Anti-PD1 antibodies (pembrolizumab and nivolumab) have shown higher response rates (ORR around 40%) and long duration of response with a more favourable toxicity profile in comparison to ipilimumab [2–4]. The combination of nivolumab and ipilimumab has shown even higher response rates (58% ORR with 19% CR) similar to the ones seen with BRAF + MEK inhibitors, but this came at a price of greater toxicity (55% of the patients on the combination regiment experience a major toxicity which usually requires hospitalization and treatment discontinuation) [4]. The full details of these pivotal trials have been reviewed elsewhere [5]. In spite of these major advances about the half the patients do not respond to immunotherapy. This has resulted in attempts to maximise the clinical benefit whilst reducing overall toxicity. In addition, there has been a major effort to identify those patients more likely to respond to immunotherapy and to exclude those unlikely to benefit. Lastly, efforts are made to convert non-responders into responders. Unfortunately most attempts to improve the clinical outcome involve combination with other agents and usually result in significant additional toxicity.

IMM-101 is a suspension of heat-killed whole cell *Mycobacterium obuense* (*M. obuense*) sourced from the National Collection of Type Cultures (NCTC) with reference NCTC 13365 in borate-buffered saline, produced in accordance with good manufacturing practice (GMP) for intradermal administration to humans. It is usually given as a single 0.1 mL intradermal injection of IMM-101 (10 mg/mL) into the skin overlying the deltoid muscle with an induction phase of 9 weeks initially and a maintenance phase thereafter. It is a non-specific immune modulator and has not shown significant systemic toxicity. It has demonstrated to induce clinical responses in stage IV melanoma patients (especially cutaneous and lung metastases) (IMM-101-001 study) as well as resulting in prolonged 5 years survival (IMM-101-008 study) in this small cohort [6, 7]. In combination with gemcitabine it has shown to significantly improve the survival outcomes of patients with metastatic pancreatic cancer, as compared to gemcitabine alone [8]. IMM-101 has been well tolerated overall in clinical studies to date, including over extended dosing periods for some patients. The only treatment-related adverse events to have occurred in more than 10% of patients when analysed across all clinical studies to date are mild pyrexia (10.5%) and injection site reactions (12.4%).

## Methods

Here we report on three cases who have received treatment with IMM-101 on the IMM-101-001 study prior to a checkpoint blockade and on a case who received nivolumab and IMM-101 concomitantly. All experienced very good response to treatment, one of which commenced within 4 days of adding pembrolizumab.

## Results

A total of 11 patients with metastatic melanoma have been treated with IMM-101 and a checkpoint inhibitor either sequentially (n = 9) or concomitantly (n = 2). Five patients experienced a disease progression, whereas the remaining patients had stability of disease at first re-assessment on treatment (n = 1) or an objective response (n = 5). Four patients had an exceptionally good and rapid response and their characteristics are summarized in Table 1.

**Table 1:** Patients’ characteristics at time of administration of IMM-101

	Patient 1	Patient 2	Patient 3	Patient 4
M1 stage AJCC v.8	M1a (0)	M1c (1)	M1c (0)	M1b (0)
Baseline LDH	Normal	Abnormal	Abnormal	Normal
BRAF status	Wild-type	Wild-type	Wild-type	Wild-type
Number of metastases	6	> 10	> 10	5
Sites of metastases	Subcutaneous	Lung, peritoneum, stomach, nodes	Lung, subcutaneous, nodes, adrenal	Lung, subcutaneous

### Patient 1

**Fig. 1:**
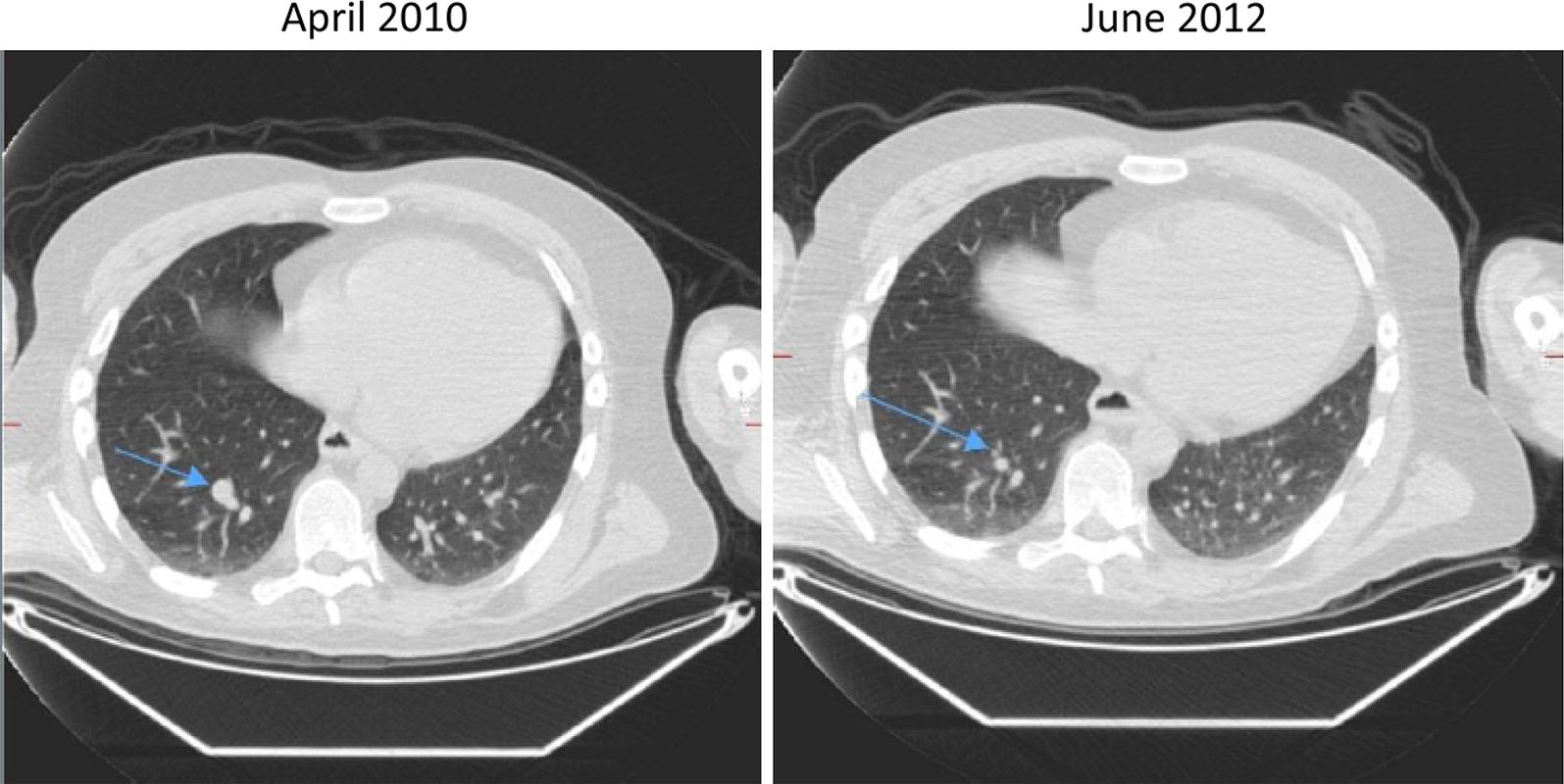
Significant response seen in a 46 year-old male with stage IV melanoma treated with ipilimumab after IMM-101 with most of the lesions resolving and a couple remaining stable over a long period of time

A 46 year old male who presented in 2006 with a 3.7 mm BT (Breslow thickness), BRAF wild-type melanoma on his left forearm. He had 1 positive lymph node at SNB (Sentinel Node Biopsy) with no further nodal involvement at left axillary dissection. He had over the years multiple subcutaneous loco-regional recurrences treated with surgical resections initially and then with topical imiquimod and intra-tumour IL-2, as per Green et al. [9]. Further recurrences led to him being enrolled on the IMM-101-001 study, which resulted in a reduction in the rate of new disease. Following the development of lung metastases he stopped IMM-101 and received 6 weeks thereafter ipilimumab on a clinical trial. On ipilimumab he experienced a rapid very good response (partial response [PR] > 50% as per RECIST 1.1 criteria) with most of the lesions resolving and a couple of visceral lesions remaining stable now over 5 years (Fig. 1).

### Patient 2

A 50 year-old female who presented in 2011 with an axillary mass, which on removal was shown to be a BRAF wild-type metastatic melanoma. No primary tumour was identified. She developed mediastinal, lung, gastric and peritoneal deposits within a couple of months from initial diagnosis. She had a partial gastrectomy to remove the cancer which was bleeding, and cyberknife treatment for the metastatic lung lesion. She also received systemic treatment with dacarbazine followed by IMM-101 on the IMM-101-001 study, which resulted in a minor response. She remained however stable for about a year until 2013 when she presented with a small bowel obstruction from new disease. She stopped IMM-101 and started ipilimumab and experienced a rapid complete response as per RECIST 1.1 criteria, which continued for 2 years until she had a further recurrence following trauma and stress in 2015. She is still disease free at the moment after further surgery.

### Patient 3

**Fig. 2:**
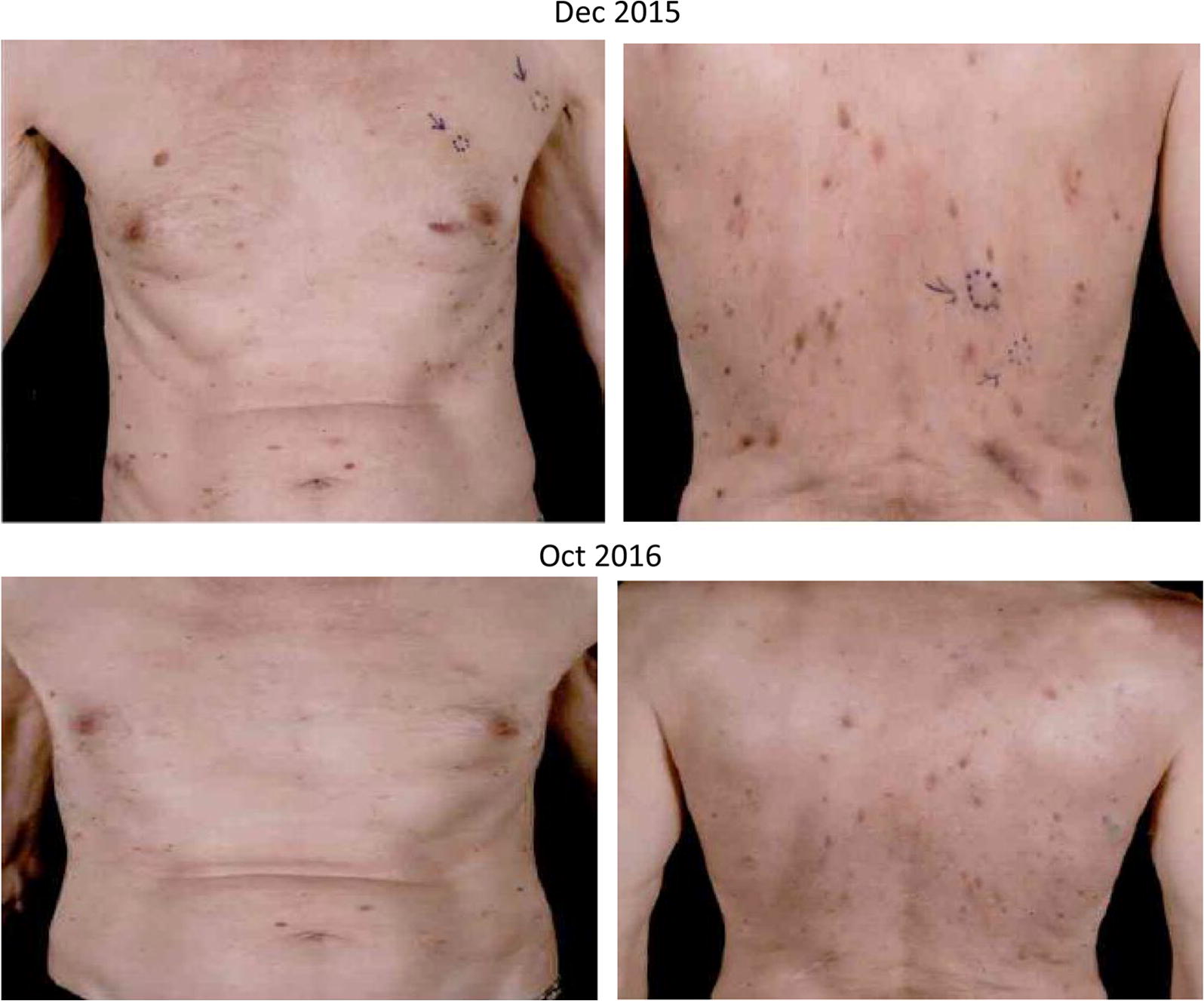
Complete reponse observed in a 79 year-old male with stage IV melanoma treated with IMM-101 followed by pembrolizumab (subcutaneous disease). The subcutaneous lesions started to shrink within 4 days of the first infusion of pembrolizumab

**Fig. 3:**
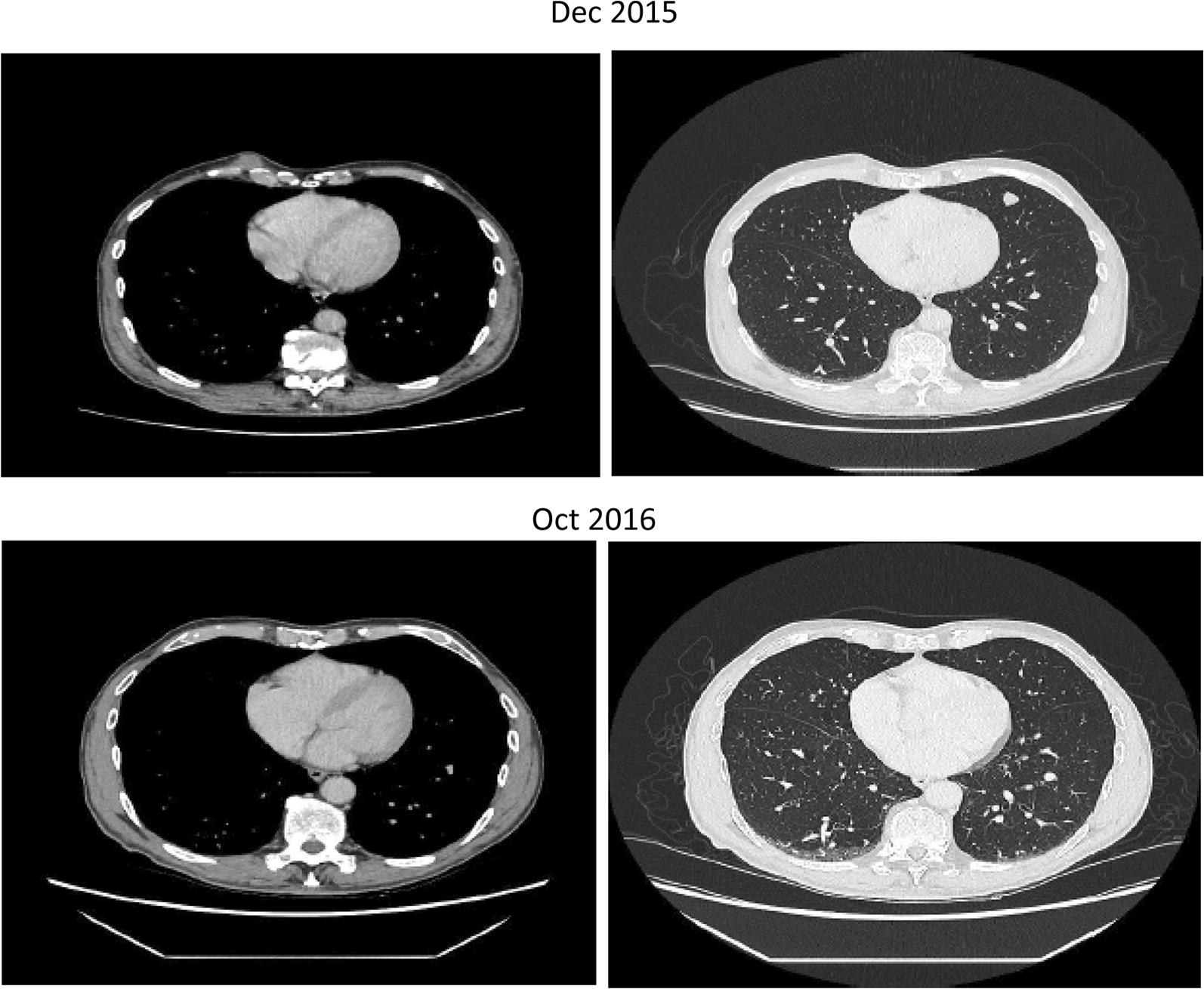
Complete reponse observed in a 79 year-old male with stage IV melanoma treated with IMM-101 followed by pembrolizumab (lung disease)

A 79 year-old male presented in 2014 with a melanoma on his left cheek (BT 2.4 mm, not ulcerated, BRAF wild-type) with a positive SNB leading to left neck dissection at time of diagnosis (no further positive nodes). Within months he developed paracardiac nodes, adrenal, lung and multiple large subcutaneous metastatic deposits. In view of his age and performance status he was commenced on IMM-101 on a named-patient program with initial stabilisation of disease. Upon progression of the subcutaneous disease he stopped IMM-101 and started with pembrolizumab, which showed a very rapid benefit as the subcutaneous lesions started to shrink within 4 days of the first infusion (Fig. 2). All visceral disease had also responded as seen on a restaging CT scan performed 6 weeks later with a PR > 50% as per RECIST1.1 criteria initially followed by a CR a few months afterwards (Fig. 3) upon continuation of pembrolizumab which lasted for 18 months and is still ongoing.

### Patient 4

A 63-year old male who presented in July 2016 with a 4.2 mm BT ulcerated BRAF wild-type nodular melanoma on his right upper back. He underwent wide local excision and SNB of his right axilla in September 2016 (N + 3/5) followed by completion of the lymphadenectomy in October 2016 (N + total 3/15). In May 2017 he developed subcutaneous metastases on the right lower leg, right forearm and anterior scalp. At the same time a re-staging CT scan showed new pulmonary disease (at least 3 metastatic nodules, the bigger measuring 1.6 cm in the larger diameter). He was then enrolled in the IMM-101-011 study aiming to evaluate the safety and efficacy of IMM-101 in combination with standard of care in patients with metastatic cancer. On the 1st of June 2016 he started treatment with nivolumab in combination with IMM-101. The combination treatment has been well tolerated with no major toxicities apart from transient hyperthyroidism. A minor response to treatment (SD as per RECIST 1.1 criteria) was observed at first re-assessment in August 2016 with a complete response achieved in December 2017. IMM-101 has been administered regularly every 4 weeks on the named-patient program after trial closure till December 2017 when he developed a grade 3 skin reaction at the site of the latest injection of IMM-101. Since then the treatment with IMM-101 has been postponed and rescheduled every 3 months.

## Discussion

IMM-101 was developed following considerable clinical experience with a similar agent, *Mycobacterium vaccae*, which was reported as inducing clinical responses in low volume metastatic melanoma, as well as enhancing the effect of other therapies [10, 11]. This led to many patients with advanced disease becoming manageable with other treatments, including surgery, resulting in a 5 years OS of over 20% in a phase II study [11]. Heat-killed *M. vaccae* was produced by SR Pharma and called SRL-172, but was eventually dropped in favour of mRNA-silencing technology when the company changed its name to Silence Therapeutics. IMM-101, based on *M. obuense* was selected by Immodulon Therapeutics as a similar but more suitable agent for clinical development. An early phase II with this agent showed a similar pattern of clinical responses, as seen with SRL-172, and increased OS (30% at 5 years) [6, 7].

IMM-101 generates an aspecific immune response and its full mechanism of action is still under investigation. Exposure of IMM-101 has shown to prime in vitro generated murine dendritic cells and human monocyte-derived DC in a dose dependent manner that functionally affects DC by enhancing their ability to process and present antigen [12, 13]. Furthermore it has been observed that IMM-101 activated DCs are able to promote CD8^+^ and CD4^+^ (Th-1) T cell secretion of IFN-γ following re-stimulation of draining lymph node cell preparations, 7 days after subcutaneous adoptive transfer of IMM-101 primed DC into naïve recipient mice [12]. In this experiment, it was not only shown that IMM-101 stimulated DCs lead to a large increase in the number of IFN-γ producing T cells, but also to a similar large increase of IFN-γ producing natural killer (NK) and γδ-T cells in the draining lymph node. In another experiment, Fowler et al. showed that IMM-101 activates γδ-T cells via cytokines produced by type 1 DCs [14]. These findings may suggest that IMM-101 through the activation of DCs is able to stimulate the formation of activated cytotoxic T cells (CTLs), NK and γδ-T cells, which are all crucial for killing cancer cells.

In a murine model of lung metastasis, IMM-101 showed a significant effect on the metastatic capacity of CT26 tumour cell injected intravenously by reducing the metastatic burden [15].

In a murine model with the breast cancer cell line EMT-6, IMM-101 showed to increase the response to anti-PD1 antibodies, resulting in a significant (i) reduction of tumour volume, (ii) increase of the intra-tumour CD8^+^ T cells/Treg ratio and (iii) increase of the IFN-γ/IL-10 ratio in spleen cells compared to anti-PD1 alone [16]. In similar mice experiments using murine pancreas and melanoma cancer cell lines it was also found that the combination treatment of IMM-101 with a CPI (both anti-PDL-1 and anti-CTLA-4 were tested) had stronger anti-tumour effects than the CPI alone (unpublished results and [17]).

In the above mentioned preclinical studies IMM-101 has shown to stimulate an immune response suppressed by the underlying tumour, which may enhance responses to other modalities of treatment. Altogether 11 patients with metastatic melanoma have been treated with IMM-101 and CPIs either sequentially or concomitantly. Despite the majority of these patients were previously pre-treated, about the half of them experienced some kind of benefit. Four of them had an exceptionally good and rapid response and have been presented here. The CR rate with ipilimumab in melanoma is less than 3%. We reported 2 cases that had both received prior IMM-101 treatment and experienced a very good response with ipilimumab; one CR which lasted for a couple of years until unexpected trauma and the other with no new disease for 5 years. Although the CR for PD-1 inhibitors is higher, complete responses may take months before they become manifest. Here we presented a case of widespread stage IV M1c disease that had, following prior IMM-101 treatment, an objective response observed 4 days after the first infusion with the anti-PD1 antibody pembrolizumab and another case of a rapid complete response to treatment when IMM-101 was given in combination with the anti-PD1 antibody nivolumab.

Based on our experience with IMM-101 (either as part of a clinical trial or a named-patient program with several different cancers) and with CPIs in melanoma, we think that the use of IMM-101 in these 4 patients may have enhanced the effects of the CPIs resulting into the very good and rapid responses seen in these patients. IMM-101 may have activated in these patients a complementary pathway which is synergistic with CPI treatment.

Using murine melanoma models, Salmon et al. [18] have shown that CD103^+^ dendritic cells (so-called cDC1 cells, a special subclass of DCs that elicit type I immune responses which are required for effective anti-tumour responses) were the only antigen presenting cells (APCs) that, following proper activation by danger signals (such as danger-associated molecular patterns (DAMPs) from dying tumour cells and pathogen-associated molecular patterns (PAMPs) from microbes [19, 20], were able of transporting tumour antigens to the lymph nodes and capable of priming there CD8^+^ T-cells and transforming them into CTLs. In these murine melanoma models, CD103^+^ DCs were required to promote the anti-tumour effects of administered anti PD-L1, which only had partial activity when used alone. Salmon et al. showed that a therapy of Fit3L (growth factor for DCs) combined with poly1:C (a PAMP that activates DCs) can enhance the anti-tumour responses of CPIs in murine models by inducing CD103^+^ DC maturation, proliferation and activation. cDC1 have the unique ability of cross presentation of (tumour) antigen epitopes embedded in class I major histocompatibility complex molecules [HLA I (mice) and MHC I (human)], which is a prerequisite for the recognition of these (tumour) antigens by CD8^+^ T-cells and their subsequent priming, maturation, activation and proliferation.

IMM-101 has been shown to activate naïve mouse and human DCs into DCs that are capable to effectively present antigens and to elicit the required type I immune response [12, 13, 16, 21] that Salmon et al. found to be required for effective CPI responses. Humans do not have CD103^+^ DCs but they do have similar (CD141^+^) cDC1-like cells capable of priming CD8^+^ T-cells when activated [22]. In humans, IMM-101 is given intradermally and it is assumed that it activates intradermal DCs (and possibly also Langerhans cells) with its PAMPs. Following activation, these DCs migrate to the lymph nodes, an activity that was readily demonstrated by PET scan following IMM-101 administration (unpublished results). As mentioned above, in mouse models, IMM-101 induces a powerful Th-1 and CTL cytokine response, which attenuates unwanted Th-2 responses and high levels of IFN-γ can be found in lymph nodes containing IMM-101 activated DCs. This IFN-γ was found to be produced not only by increased numbers of Th-1 and CTLs, but also by similar increased numbers of activated NK and γδ-T cells, which are all cells that, after traveling to the tumour, are together required for a highly effective anti-tumour response. It is this systemic type I immune modulating activity that suggests that the human equivalent of CD103^+^ DCs may be activated by intra-dermally injected IMM-101, leading to the enhancement of CPI responses. Clearly further work to confirm the cDC1 activation as the dominant explanation of the observed synergy between IMM-101 and CPIs are needed.

## Conclusion

Our positive experiences with these 4 patients and the absence of any severe systemic toxicity clearly warrants in our view a pilot clinical trial to assess the safety and the efficacy of IMM-101 in combination with nivolumab in metastatic melanoma patients. Such a trial (IMM-101-015) is expected to start mid-2018.
